# The pregnancy outcomes of patients with rudimentary uterine horn: A 30-year experience

**DOI:** 10.1371/journal.pone.0210788

**Published:** 2019-01-25

**Authors:** Xiaoyan Li, Ping Peng, Xinyan Liu, Weilin Chen, Juntao Liu, Jianqiu Yang, Xuming Bian

**Affiliations:** Department of Obstetrics and Gynecology, Peking Union Medical College Hospital, Beijing, China; University of Insubria, ITALY

## Abstract

**Objectives:**

To evaluate the presentation, assessment, treatment, and pregnancy outcomes of 22 women with a rudimentary uterine horn.

**Methods:**

We reviewed the data regarding the outcomes of patients with a rudimentary horn pregnancy (RHP) who were managed at Peking Union Medical College Hospital over the last 30 years. Twenty-two pregnant patients with a rudimentary horn have been treated at our institute over the last 30 years. All patients with RHP were divided into two groups: Type A (n = 4), a rudimentary horn with a cavity that communicated with the uterus; and Type B (n = 7), a rudimentary horn with a cavity that did not communicate with the uterus. We classified all 22 patients into communicating group or noncommunicating group according to the anatomical connection of the rudimentary horn to the contralateral hemiuterus.

**Results:**

The mean gestational age of Type A patients (23.5 weeks) was significantly higher (*P* = 0.046) than that of Type B patients (10 weeks). The rudimentary uterine horn carried 4 of 5 (80%) pregnancies in the communicating group. Three case of rudimentary horn pregnancies ruptured before a gestational age of 12 weeks, and one abortion occurred after a gestational age of 12 weeks. In the noncommunicating group, 7 of 17 (41.2%) cases were RHPs, and 3 ruptured after a gestational age of 12 weeks.

**Conclusions:**

The diagnosis and management of the rudimentary uterine horn continues to be challenging. Medical and radiological personnel must maintain a high degree of alertness to prevent the morbidity associated with this condition. In particular, patients with RHP (Type A), who have a higher chance being misdiagnosed before 12 gestational weeks, have a higher risk of potential complications. If pregnancy in the rudimentary horn is diagnosed, excision of the pregnant horn is recommended, regardless of the type of unicornuate uterus.

## Introduction

Mullerian abnormalities are present in 0.17% of fertile women and 3.5% of infertile women, and unicornuate uterus is observed in 0.4% of women[1–3]. Approximately 84% of unicornuate uteruses have a contralateral rudimentary horn[4].

An embryo can implant in a uterus with a rudimentary horn or in a unicornuate uterus. Although these conditions are similar, their reproductive outcomes are completely different.

Rudimentary horn pregnancy (RHP) is rarer still, with a reported incidence ranging from 1 in 76,000 to 1 in 150,000[5]. RHP results in the rupture of the horn by the second trimester in 80–90% of all cases. Only 14% of all cases are diagnosed before clinical symptoms occur[6]. Most cases of RHP provide a diagnostic challenge and are diagnosed after rupture, which leads to emergency surgery, blood transfusions, and increased morbidity[7–13]. Early diagnosis before rupture is essential for the successful management and prevention of maternal morbidity and mortality. The reproductive outcomes of women with unicornuate uteruses are poor; the associated live birth rate is only 29.2% and the prematurity rate is 44%[14, 15]. Moreover, women with this anomaly present spontaneous abortion rates of 24.3% in the first trimester and 9.7% in the second trimester[1].

A lack of published data on unicornuate uterus pregnancy and RHP exists in the medical literature, and most of the available studies are case reports[1, 13, 15–18]. A limited number of studies have reported the clinical characteristics and reproductive differences between the subtypes of rudimentary horn according to the American Fertility Society classification (AFSC). The current study aimed to describe the presentations, assessments, treatments, and pregnancy outcomes of 22 women with a rudimentary horn, and we compared the reproductive performance of these groups.

## Materials and methods

We reviewed the hospital data regarding the pregnancy outcomes of patients with a rudimentary horn who were managed at our institute, the Peking Union Medical College Hospital, over the last 30 years. Twenty-two pregnant patients with a rudimentary horn who presented at our institute from January 1, 1986, to December 31, 2016, were enrolled in the present study. We conducted this study on April 30, 2017. Patient age, gravidity, parity, diagnosis before pregnancy, abnormal gestational history, surgery history, estimated blood loss, gestational weeks, abdominal pain and vaginal bleeding at presentation, and abdominal bleeding were recorded. Abdominal pain defined as severe,acute pain of abdomin, and it is one of the main complaints of patients. Uterine anatomy was evaluated using a routine vaginal or abdominal ultrasound before or after the patient became pregnant. Some patients were underwent hysterosalpingography, laparoscopy or were diagnosed by macroscopic detection during cesarean section. Patients with RHP were managed by excision of the rudimentary horn combined with ipsilateral salpingectomy. Patients with a unicornuate uterus pregnancy were diagnosed with unicornuate uterus pregnancy featuring a contralateral rudimentary horn upon cesarean section. Patients with RHP were divided into the following two groups according to the AFSC classification:

### Type A (n = 4)

A rudimentary horn with a cavity that communicates with the uterus.

### Type B (n = 7)

A rudimentary horn with a cavity that does not communicate with the uterus.

The other 11 patients with a unicornuate uterus pregnancy were definitively diagnosed with a rudimentary uterine horn during cesarean section. If the horn did not communicate with the main cavity of the contralateral hemiuterus, then we were unable to confirm that it contained a functional endometrium. Thus, we classified all 22 patients into communicating group or noncommunicating group according to the anatomical connection of the rudimentary horn to the contralateral hemiuterus (see S1 Table). The local ethical committees of Peking Union Medical College Hospital granted a waiver of approval for this retrospective study, and patients provided informed consent for the use of their medical records in retrospective studies prior to surgery.

Nonnormally distributed continuous variables were compared using the Mann-Whitney U test and the Kruskal-Wallis test. Two-tailed *P*-values are reported, and the alpha for all tests was set to 0.05. All statistical analyses were performed using SPSS 12.0 (SPSS, Inc., Chicago, IL, USA).

## Results

Table 1 compares the characteristics and clinical data from Type A and Type B patients. The mean gestational age of Type A patients (23.5 weeks) was significantly higher (*P* = 0.046) than that of Type B patients (10 weeks). Type A patients exhibited higher frequencies of abdominal pain (100% vs 42.9%) than did Type B patients, but the difference was not statistically significant (*P* = 0.071). Mean age, gravidity, parity, ipsilateral renal agenesis, intra-abdominal hemorrhaging, and the rate of diagnosis before pregnancy were not significantly different between the two types.

**Table 1:** Characteristics and clinical data of Type A and B patients.

Clinical data	Type A	Type B	*P* value
	n = 4	n = 7	
Age (years)	26 (23–27)	29 (24–32)	0.154
Median gravidity	2 (1–3)	1 (1–2)	0.210
Median parity	0 (0–1)	0 (0–1)	0.673
Gestational age (weeks)	23.5 (10–33)	10 (7–17)	0.046
Ipsilateral renal agenesis	1/4 (25%)	1/7 (14.3%)	0.683
Diagnosed before pregnancy	0/4 (0%)	1/7 (14.3%)	0.450
Vaginal bleeding	1/4 (25%)	1/7 (14.3%)	0.673
Abdominal pain	4/4 (100%)	3/7 (42.9%)	0.071
Rupture/Intra-abdominal hemorrhaging	3/4 (75%)	3/7 (42.9%)	0.326

Table 2 shows the baseline characteristics of the patients with communicating and noncommunicating rudimentary uterine horns. Of the 22 patients, the mean age of patients in the noncommunicating group (29 years) was significantly higher (*P* = 0.036) than the mean age of patients in the communicating group (26 years). Patients in the communicating group exhibited significantly higher frequencies of abdominal pain (100% vs 17.6%) than did the patients in the noncommunicating group, which is consistent with the results shown in Table 1. Gravidity, parity, gestational age, ipsilateral renal agenesis, abnormal gestational history, surgery history, infertility history, intra-abdominal hemorrhaging, and the rate of RHP diagnosis before pregnancy were not significantly different between the two groups.

**Table 2:** Characteristics and clinical data for patients with communicating and noncommunicating rudimentary uterine horns.

Clinical data	Communicating	Noncommunicating	*P* value
	n = 5	n = 17	
Age (years)	26 (23–27)	29(24–38)	0.036
Median gravidity	2 (1–3)	1 (1–3)	0.078
Median parity	0 (0–1)	0 (0–1)	0.907
Gestational age (weeks)	24 (10–37)	36 (7–41)	0.529
Vaginal bleeding	1/5 (20%)	1/17 (5.9%)	0.346
Abdominal pain	5/5 (100%)	3/17 (17.6%)	0.001
Ipsilateral renal agenesis	2/5 (40%)	3/17 (17.6%)	0.476
Abnormal gestational history	1/5 (20%)	1/17 (5.9%)	0.346
Surgery history	1/5 (20%)	4/17 (23.5%)	0.872
Infertility history	0/5 (0%)	6/17 (35.3%)	0.128
Diagnosed before pregnancy	1/5 (20%)	11/17 (64.7%)	0.085
RHP	4/5 (80%)	7/17 (41.2%)	0.136
Rupture/Intra-abdominal hemorrhaging	3/5 (60%)	3/17 (17.6%)	0.068

The reproductive outcomes of the 22 patients are shown in Table 3. The rudimentary uterine horn carried 4 of 5 (80%) pregnancies in the communicating group. Three pregnancies ruptured before a gestational age of 12 weeks, and one abortion occurred after 12 weeks. In the noncommunicating group, 7 of 17 (41.2%) patients presented with RHPs, and 3 pregnancies ruptured after a gestational age of 12 weeks. The remaining 4 unruptured pregnancies were diagnosed and managed before 12 weeks. However, the number of patients was too small to obtain a significant difference between two groups.

**Table 3:** Reproductive performance of patients with communicating and noncommunicating rudimentary uterine horns.

	Communicating	Noncommunicating	*P* value
	(n = 5)	(n = 17)	
Rudimentary horn pregnancy	4 (80%)	7 (41.2%)	0.136
Ruptured	3 (60%)	3 (17.6%)	0.071
Unruptured	1 (20%)	4 (23.5%)	
Early abortion			
Ruptured	1 (20%)		
Unruptured		4 (23.5%)	
Late abortion			
Ruptured	1 (20%)	3 (17.6%)	
Unruptured	1 (20%)	/	
Preterm delivery			
28–37 weeks	1 (20%)		
Unicornuate uterus pregnancy	1 (20%)	10 (58.8%)	0.136
Preterm delivery			
28–37 weeks		1 (5.9%)	
Term delivery			
PROM		2 (11.8%)	
Neonatal malformation (foot inversion)		1 (5.9%)	
Oligohydramnios		1 (5.9%)	
Breech presentation		3 (17.6%)	
Normal delivery	1 (20%)	2 (11.8%)	

Six patients suffered rupture of the pregnant horn with massive intra-abdominal hemorrhaging and shock. The mean intra-abdominal blood loss volume was 2,600±418.33 ml. All of these patients received multiple blood transfusions.

In the patient who presented with an abdominal pregnancy, the pregnancy was ultimately confirmed as having developed from a ruptured rudimentary uterine horn. We performed laparotomy at 33 gestational weeks and observed a fragile placenta located in the ruptured rudimentary uterine horn, and a living neonate was retrieved from the peritoneum. The neonate weighed 1,855 g and had 1-minute and 5-minute Apgar scores of 10 and 10, respectively. The neonate had no gross congenital abnormalities.

Eleven unicornuate uterus pregnancies were recorded, and 10 reached term. Only one preterm delivery occurred in the communicating group. This neonate weighed 1,150 g and had 1-minute and 5-minute Apgar scores of 10 and 10, respectively. A cesarean section was performed due to the preterm premature rupture of membrane (PPROM), an elevated C-reactive protein level and the patient’s firm request.

The noncommunicating group included 9 neonates delivered at term. Certain maternal and fetal abnormalities were observed, including one neonate with macrosomia weighing 4,190 g, one neonate with oligohydramnios, one preterm delivery with premature rupture of membranes (PROM), one case of PROM, and one neonatal malformation (foot inversion). Overall, one-third of these pregnancies involved a breech presentation.

## Discussion

Unicornuate uteruses are further subdivided into 4 variants, according to the criteria from the American Fertility Society[19]. Isolated unicornuate uteruses are the most common type, with a reported frequency of 35%. When a rudimentary horn is present, it is the noncavitary type in 33% of cases, the cavitary but noncommunicating type in 22% of cases, and the cavitary and communicating type in 10% of cases[20, 21].

We classified all 11 RHPs into two types (Type A and Type B) according to the AFSC criteria. The gestational age of Type A patients (mean gestational age>12 weeks) was significantly higher than that of Type B patients (mean gestational age<12 weeks). The possible explanation for this discrepancy might be the fact that a contralateral rudimentary horn with communicating cavity is closer to a pregnancy in a normal uterus. Thus, the diagnosis is more difficult and is frequently delayed until the patients have symptoms or the horn has even ruptured. Based on this finding, the risk of misdiagnosis of RHP with a communicating horn is higher, and these patients should be more carefully monitored if they become pregnant. Meanwhile, the broadly attached rudimentary horn is likely to receive its blood supply not only from the ipsilateral uterine artery but also from the myometrial arcuate arteries of the contralateral uterine artery[22]. Therefore, the vascularization of the rudimentary horn in Type A patients is more extensive than that in Type B patients, and the muscle of the rudimentary horn is thicker in Type A patients than in Type B patients, which lead to the higher gestational age of Type A patients. The higher gestational age of Type A may also lead to more frequently abdominal pain (the ratio of abdominal pain in Type A was 100%). Regarding the unicornuate uterus pregnancy, most were diagnosed during a cesarean section, and a noncommunicating rudimentary horn was identified. Although the exact classification of a functional endometrium in noncommunicating horns cannot be determined during surgery, we classified all 22 patients into a communicating group and a noncommunicating group. Significant differences in the RHP rate or uterine rupture rate were not observed between the two groups. However, all three rupture cases in the noncommunicating group occurred during the second trimester. The patients did not undergo any other examination during pregnancy except a urine HCG test until they visited the emergency department in our hospital. Meanwhile, the remaining four unruptured cases all occurred in the first trimester. Two patients were diagnosed with an ectopic pregnancy and uterus didelphys, respectively. Two other patients were diagnosed with RHP. We postulate that the noncommunicating rudimentary uterine horn was more distinct from a unicornuate uterus, and patients might have received an earlier diagnosis if an ultrasound was performed during the first trimester.

If a woman suspected of having a rudimentary horn becomes pregnant, and the pregnancy may be located in the rudimentary horn, close monitoring is warranted because of the risks of rupture and its complications. Although neonates have survived RHPs, life-threatening uterine rupture during the early or mid-gestational ages remains the most likely outcome, and neonatal survival remains rare. We stress the need for an accurate diagnosis before pregnancy, a proper consultation and a quick surgical treatment only in these severe and rare cases. Early diagnosis before rupture is essential for the successful management and prevention of maternal morbidity and mortality.

Cases of late and false diagnoses leading to uterine rupture have been reported repeatedly in the literature[7–13]. Some patients were diagnosed only after an attempt to evacuate the uterus to terminate an incorrectly diagnosed intrauterine pregnancy[23–25]. We found few published cases of early (first trimester) prerupture sonographic diagnoses of this condition[26, 27]. Most case studies describe patients who were either symptomatic or known to have a uterine abnormality. Thus, the early diagnosis of RHP remains challenging. In our case series, only one of 11 patients with RHPs was referred with a known noncommunicating rudimentary horn, but she did not undergo surgery to remove her rudimentary horn uterus. The other 10 patients received a false or misdiagnosis, with 3 suspected diagnoses: ectopic pregnancy in one patient, uterus didelphys in three patient, gestational trophoblastic disease in one patient, and appendicitis in two patients made by a general surgeon. Pregestational diagnoses were not available for most of the patients; thus, they ultimately presented with a rupture of the rudimentary uterine horn. All 11 cases with a unicornuate uterus pregnancy were diagnosed with uterus didelphys and a unicornuate uterus with or without a rudimentary uterine horn before pregnancy. Among these patients, four patients were diagnosed by hysterosalpingography, two by ultrasound and five by laparoscopy. The reproductive performance of women with unicornuate uteruses is poor [1]. However, a successful pregnancy is possible when the diagnosis is confirmed before pregnancy. In all 11 patients diagnosed before pregnancy, 6 patients were performed with hysterosalpingography due to infertility history (Table 2). The infertility history included no case in communicating group (0/5) and 35.3% (6/17) in noncommunicating group. No patients recorded any other reason of infertility such as male factor. Although there was no statisticant significance, this difference may be attributed to the anatomical variations between two groups. The reproductive outcomes of the unicornuate uterine pregnancies in our study were acceptable; 5 of 11 patients had completely normal deliveries, and an additional 6 patients did not experience severe adverse maternal or neonatal outcomes.

Although ultrasonography and MRI are currently the most accurate prenatal diagnostic methods, the level of evidence in the literature regarding the efficacy of radiological diagnoses of RHP is low. In most cases, the initial radiological assessment leads to an incorrect diagnosis, particularly when patients present with emergency conditions. The ultrasonographic sensitivity for diagnosing RHP is low, ranging from 29–33%[4, 28]. In addition, 3D ultrasound and MRI may help with the diagnosis[29]. Tsafrir et al. proposed three criteria for an ultrasonographic diagnosis of RHP[30]. The pregestational diagnosis of a rudimentary horn requires hysterosalpingography, hysteroscopy and laparoscopy, whereas prenatal diagnoses are attempted using ultrasonography, particularly transvaginal ultrasonography[28, 31]. However, the most important factor is the experience and skill of the radiologist and his or her awareness of uterine malformations[16].

In addition to radiological indications, clinical symptoms are important. Of the 11 patients with an RHP in our study, the most common symptom (i.e., pain in the abdomen) was observed in 63.6% of patients. Signs of rupture, peritonitis, and an acute abdomen with increasing abdominal fluid were noted in 54.5% of patients who experienced rupture. Abdominal pain occurred in all 5 patients (100%) in the communicating group in our study. Therefore, pregnant patients with abdominal pain and free fluid during their second trimester should be carefully examined, particularly patients with a known diagnosis of a unicornuate uterus.

Laparoscopic removal of an accessory uterine horn (in the nonpregnant state) was first reported by Nezhat et al. in 1994[32], and several case reports have subsequently been published[33]. When the diagnosis was obtained, at least three valid reasons can justify the surgical excision of the cavitated noncommunicating rudimentary horn: removal of the cause of dysmenorrhea, the prevention of possible endometriosis caused by transtubal menstrual reflux, and avoiding an intracornual pregnancy implantation[22, 34].

The removal of a pregnant uterine horn has been reported previously[35]. As a rudimentary horn with a functional endometrium is at risk of dysmenorrhea, infertility or ectopic pregnancy, resection is recommended when it is diagnosed[36, 37]. Importantly, the risk of rupture of a pregnant rudimentary uterine horn in the second trimester is very high[10], and therefore, if pregnancy in the rudimentary horn is diagnosed, excision of the pregnant horn is recommended, although sporadic pregnancies producing a living infant, as observed for one patient in the present study[31, 35, 38]. An RHP can cause heavy bleeding and threaten the patient’s life. To date, the surgical approach for rudimentary horn uterus is excision. Other novel approaches for an obstructed hemiuterus have been described in a case report[39].

## Conclusions

The diagnosis and management of the rudimentary uterine horn remains challenging. Many women with a rudimentary uterine horn present with acute uterine rupture during pregnancy. Early diagnosis is the key to successful management. Medical and radiological personnel must maintain a high degree of alertness to prevent the morbidity associated with this condition. In particular, patients with RHP (type A), who have a higher chance being misdiagnosed before 12 gestational weeks, have higher risks of potential complications. If an RHP is diagnosed, excision of the pregnant horn is recommended because the risk of rupture of an RHP in the second trimester is very high, regardless of the type of unicornuate uterus.

## Supporting information

S1 Table Data of all pregnant patients with rudimentary uterine horn. (XLSX)
